# Urban Area Disadvantage and Under-5 Mortality in Nigeria: The Effect of Rapid Urbanization

**DOI:** 10.1289/ehp.0901306

**Published:** 2010-02-10

**Authors:** Diddy Antai, Tahereh Moradi

**Affiliations:** Division of Epidemiology, Institute of Environmental Medicine, Karolinska Institute, Stockholm, Sweden

**Keywords:** multilevel modeling, Nigeria, rapid urbanization, under-5 mortality, urban area disadvantage, urban context

## Abstract

**Background:**

Living in socioeconomically disadvantaged areas is associated with increased childhood mortality risks. As city living becomes the predominant social context in low- and middle-income countries, the resulting rapid urbanization together with the poor economic circumstances of these countries greatly increases the risks of mortality for children < 5 years of age (under-5 mortality).

**Objective:**

In this study we examined the trends in urban population growth and urban under-5 mortality between 1983 and 2003 in Nigeria. We assessed whether urban area socioeconomic disadvantage has an impact on under-5 mortality.

**Methods:**

Urban under-5 mortality rates were directly estimated from the 1990, 1999, and 2003 Nigeria Demographic and Health Surveys. Multilevel logistic regression analysis was performed on data for 2,118 children nested within data for 1,350 mothers, who were in turn nested within data for 165 communities.

**Results:**

Urban under-5 mortality increased as urban population steadily increased between 1983 and 2003. Urban area disadvantage was significantly associated with under-5 mortality after adjusting for individual child- and mother-level demographic and socioeconomic characteristics.

**Conclusions:**

Significant relative risks of under-5 deaths at both individual and community levels underscore the need for interventions tailored toward community- and individual-level interventions. We stress the need for further studies on community-level determinants of under-5 mortality in disadvantaged urban areas.

Although it has long been known that there is a correlation between individual-level socioeconomic position (SEP) and childhood mortality (Galobardes et al. 2006; Lawlor et al. 2006; Power et al. 2005), researchers have turned their attention to the role of socioeconomic characteristics of areas in child survival (Kawachi and Berkman 2003; Macintyre et al. 2002). The evidence suggests that living in socioeconomically disadvantaged areas is associated with increased mortality risks, even after adjusting for individual demographic and socioeconomic characteristics (Bosma et al. 2001; Marinacci et al. 2004; Martikainen et al. 2003; Pickett and Pearl 2001; Sundquist et al. 1999). The world’s urban population is growing at a fast pace, necessitating greater emphasis on the association between area-based measures of SEP within urban areas and the health of populations living in those areas (Eames et al. 1993; Galea and Vlahov 2005). These area-based measures are seen largely as aggregate correlates of the individual measures and generally show strong graded associations to most health outcomes. This not only mimics the associations seen at the individual level (Kaplan 1996) but also reflects the health effects of physical and social infrastructure above and beyond individual compositional effects (Kaplan 1996; Macintyre et al. 1993).

Half of the world’s population (3 billion people) now live in urban areas, and it is expected that by 2030 about two-thirds of the world’s population (5 billion people) will live in urban areas (United Nations Department of Economics and Social Affairs 2004).

Urbanization, the process of becoming urban, reflects aggregate population growth in cities through either natural population increase or migration (Galea and Vlahov 2005) and is inextricably linked with development. As a result, urban or city living has become the ideal for many people in low- and middle-income countries (Kasarda and Crenshaw 1991). Urban living has important health benefits, such as better access to health care, education, and social amenities (McMichael 2002; Vlahov and Galea 2002). However, with the present pace of urbanization in low- and middle-income countries such as Nigeria and within the context of poor economic performance, poor governance, failure of national and urban housing policies, and institutional and legal failure, the capacity of most urban economies in developing countries is overstretched. Hence, only a fraction of the growing social needs of urban areas are met (United Nations Human Settlements Programme 2003), resulting in an increasing proportion of urban dwellers living under disadvantaged conditions that are characterized by overcrowded or deteriorating housing, inadequate social amenities, and poor environmental and sanitary conditions, as well as poor economic opportunities. This in turn increases the susceptibility of residents in these areas to a variety of health problems and increases childhood mortality risks (Alexander and Ehrlich 2000; Galea and Vlahov 2005; Gracey 2002; Hembree et al. 2005; Krieger and Higgins 2002; McMichael 2002; Northridge and Sclar 2003; Popkin 2001; Satterthwaite 2000; Vorster 2002; Zulu et al. 2002). Under such disadvantaged conditions, the health risks arising from living in disadvantaged urban areas rival or exceed those of rural areas, despite the generally easier access of urban residents to modern health services (African Population and Health Research Center 2002; Timæus and Lush 1995), thereby outweighing the advantages of living in urban areas (Rakodi 1997).

*Why focus on urban area disadvantage?* The importance of access to safe drinking water and housing structure quality, particularly in urban areas, is well documented. Diarrhea and other infectious diseases remain the major causes of death among children < 5 years of age (Bryce et al. 2005; Fotso et al. 2007; Woldemicael 2000). Availability of these resources is highly correlated with household SEP, which in turn is influenced by poverty and overall economic development in the community. Poor and disadvantaged urban populations are characterized by overcrowding, shortage of safe water, lack of adequate waste and sanitary services, and higher levels of air pollution and other hazardous substances, which result in increased risks of infectious diseases and mortality (Mintz et al. 2001; Van de Poel et al. 2007). The SEP of people living in poor and disadvantaged urban communities is generally low and characterized by unemployment and underemployment (Ahmad et al. 2000). In addition, ownership of fewer assets and lack of access to economic resources among people living in poor and disadvantaged urban communities make them less able to cope with ill health (Adepoju 2004; Kandala et al. 2006; Wichmann and Voyi 2006). This is the urban neighborhood context in which a large number of residents of densely populated areas live in Nigeria. Furthermore, although the spatial concentration of poverty is essential to the definition of disadvantaged neighborhoods, current efforts at systematizing this definition use indicators such as access to safe drinking water, adequate sanitation, electricity, overcrowding, and security of housing tenure. The focus is often on households rather than directly taking into account the concentrations of poverty or affluence in the neighborhoods that surround these households. Neighborhood effects are a leading example of the forces operating outside households that can exert influence on household-level behavior and health outcomes (Montgomery and Hewett 2004). Thus, there is ample reason, on both substantive and methodological grounds, to explore neighborhood effects of the urban areas of low- and middle-income countries.

Nigeria had possibly the fastest urbanization growth rate in the world in the 1970s (Akinbame and Fadare 1997). Between 1970 and 1980, the proportion of Nigerians living in urban areas was estimated to have grown from 16% to > 20%, and by 2010, urban population is expected to be > 40% of the nation’s total (Akinbame and Fadare 1997). In 1995 Lagos (the former administrative capital of Nigeria) was the world’s 29th largest urban agglomeration, with 6.5 million inhabitants, and in 2000 it became the 23rd largest, with 8.8 million people. In 2002, Lagos became one of sub-Saharan Africa’s first mega-urban regions with its metropolitan population reaching 10 million inhabitants. The city continues to grow, and by 2015 it is expected to become the world’s 11th largest urban system, with 16 million inhabitants (United Nations Human Settlements Programme 2003).

As city living becomes the predominant social context for most of the world’s population, the urban environment is bound to shape population health in cities (Galea and Vlahov 2005). Thus, explaining the association between urban area disadvantage and mortality for children < 5 years of age (under-5 mortality) in low- and middle-income countries undergoing rapid urbanization is of importance in developing appropriate health interventions and preventive measures for the rising number of urban inhabitants.

## Under-5 mortality

The under-5 mortality rate is a leading indicator of the level of child health and overall development in countries (McGuire 2006). As such, it is an indicator of the Millennium Development Goals (United Nations 2008), which seeks to reduce the under-5 mortality rate by two-thirds between 1990 and 2015. Under-5 mortality measures child survival and reflects the impact of social, economic, and environmental circumstances as well as other causes of death on infants, toddlers, and young children, including their health care (United Nations Children’s Fund 2007; United Nations Population Fund 2003). Thus, the under-5 mortality rate captures > 90% of the global mortality among children < 18 years of age (United Nations Children’s Fund 2008) and shows large variation across socioeconomic groups and geographic areas and between rural and urban areas. Moreover, data on under-5 mortality are relatively reliable compared with other measures of population health (United Nations Population Fund 2003).

We used a multilevel approach to account for the hierarchical structure of the Demographic and Health Survey (DHS) (National Population Commission 2004) data—data for children (level 1) who were clustered within data for mothers (level 2), who were in turn clustered within data for communities (level 3)—because of its suitability for investigating the relationship between area-level socioeconomic disadvantage and mortality using census data or survey data (Bosma et al. 2001; Diez-Roux 2001, 2004; Subramanian 2004). This is based on the notion that area-level characteristics are potential determinants of health outcomes and that area-level inequalities may be relevant in the context of increasing geographic clustering of poverty with other forms of disadvantage (Gephart 1997). Although several studies have assessed child survival in urban areas of sub-Saharan Africa, this study is unique in its assessment of the effect of urban area/neighborhood socioeconomic disadvantage on under-5 mortality.

The aims of this study were *a*) to assess the trend of urban under-5 mortality in relation to urban population growth in Nigeria and *b*) to assess whether area-level socioeconomic disadvantage has an impact on under-5 mortality risks after individual demographic and socioeconomic characteristics are taken into account.

## Methods

Cross-sectional data from the 2003 Nigeria DHS were used in this study. This sample was collected using a stratified two-stage cluster sampling procedure. A full report and detailed description of the data collection procedures are presented elsewhere (National Population Commission 2004). Birth history data, such as sex, month and year of birth, survivorship status, and current age or age at death if the child had died were all collected for each of these births. This study was restricted to children born to the subsample of 2,118 mothers living in urban areas at the time of the survey and to births in the last 5 years before the survey to ensure that the household variables investigated provided a close enough or accurate picture of the current living conditions of the children within the period they were exposed to increased risks of mortality.

### Ethical considerations

This study is based on analysis of secondary data with all participant identifiers removed. The survey was approved by the National Ethics Committee in the Federal Ministry of Health of Nigeria and the Ethics Committee of the Opinion Research Corporation Macro International, Inc. (ORC Macro Inc., Calverton, MD, USA). Permission to use the DHS data in this study was obtained from ORC Macro Inc.

### Measures

#### Outcome variable

The outcome variable was the risk of under-5 mortality, defined as a child dying between birth and the fifth birthday. Under-5 mortality was estimated for the 5 years preceding the survey. All children born within the 5 years before the survey date were included in the analysis. Children contributed person-time until they reached 60 months of age or until death or the date of the survey. All deaths among children ≤ 59 months were regarded as cases.

#### Exposure variables

Urban area disadvantage was measured using the urban area disadvantage index (UADI) score. The UADI scores reflect the overall level of urban area disadvantage based on eight indicators of socioeconomic disadvantage at the neighborhood level, including the percentage of children *a*) living in a household without piped water, *b*) living in a household without flush toilet, *c*) living in a household without electricity, *d*) living in a household without nonpolluting cooking fuel, *e*) whose mothers were unemployed, *f*) whose mothers were uneducated, *g*) living in crowded households, and *h*) living in households within the lowest two wealth quintiles (poorest 40%).

The UADI scores were generated through principal component analysis at the level of primary sampling units (PSUs). We included 165 urban PSUs in this study out of a total of 365. PSUs or clusters are administratively defined areas used as proxies for “neighborhoods” or “communities” (Diez-Roux 2001). These are small, fairly homogeneous units made up of one or more enumeration areas, which are the smallest geographic units for which census data are available in Nigeria. Each cluster consisted of a minimum of 50 households, with contiguous enumeration areas added when a cluster had < 50 households (National Population Commission 2004).

A similar index has been used in other studies (Barnes et al. 2007; Noble et al. 2006) in the following situations: *a*) when the main focus of analysis lies in the effects of characteristics of place of residence on health (Macintyre et al. 2002; Whitley et al. 1999), *b*) to allow for the control of possible socioeconomic confounding when examining the effects of the local environment on health (Macintyre et al. 2002), and *c*) when data describing an individual’s socioeconomic circumstances have not been, or cannot be, collected directly (Danesh et al. 1999). The clusters were ranked on the basis of the continuous UADI scores and categorized into quintiles divided at the 20th, 40th, 60th, and 80th percentiles, such that class I was assigned to the 20% least disadvantaged urban areas and class V the 20% most disadvantaged urban areas. The ranks indicate how a neighborhood compares with all the other neighborhoods and are easily interpretable. Normalized sample weights provided in the DHS data were used for this analysis, employing Stata version 10 (StataCorp, College Station, TX, USA) to adjust for nonresponse and enable extrapolation of findings to the general population.

#### Individual-level explanatory factors

Potential confounders were grouped into child- and mother-level demographic and socioeconomic characteristics and included *a*) sex of the child, categorized as male and female; *b*) birth order and interval between births, created by merging “birth order” and “preceding birth interval,” classified as first births, birth order 2–4 with short birth interval (< 24 months), birth order 2–4 with medium birth interval (24–47 months), birth order 2–4 with long birth interval (≥ 48 months), birth order ≥ 5 with short birth interval (< 24 months), birth order ≥ 5 with medium birth interval (24–47 months), and birth order ≥ 5 with long birth interval (48 months); *c*) mother’s age, grouped as 15–18, 19–23, 24–28, 29–33, and ≥ 34 years of age; *d*) marital status, categorized as single, married, and divorced; *e*) mother’s education, categorized as no education, primary, and secondary or higher education; *f*) mother’s occupation, grouped as professional/technical/managerial, clerical/sales/services/skilled manual occupations, and not working; and *g*) wealth index, categorized into quintiles as poorest, poorer, middle, richer, and richest.

### Statistical analyses

#### Trend in urban under-5 mortality rates between 1986 and 2003

The probability of child deaths among children < 5 years of age was directly estimated from the 1990, 1999, and 2003 Nigeria DHS birth history data. The urban population pattern for 1983–2003 was derived from the United Nations Department of Economics and Social Affairs (2004).

#### Multilevel logistic regression modeling

The data were analyzed using MLwiN version 2.10 (Rashbash et al. 2008). We fitted a multilevel model with binomial, penalized quasi-likelihood procedures and second-order linearization (Goldstein 2003). We used a three-level multilevel logistic regression analysis with 2,118 children (level 1), nested within 1,350 mothers (level 2), who were in turn nested within 165 communities (level 3). Four sequential models were fitted to *a*) examine the effect of no predictor variables in the fixed part, but only the intercepts in the random part so as to present a baseline for comparing the magnitude of contextual variations in under-5 mortality risks in subsequent models (model 0); *b*) examine the association between under-5 mortality and urban area disadvantage (model 1); *c*) adjust for child-level characteristics (model 2); and *d*) simultaneously adjust for urban area disadvantage and both child- and mother-level characteristics (model 3).

The measures of association (fixed effects) for each of these models were expressed as odds ratios (ORs) and their 95% confidence intervals (CIs). Measures of variation (random effects) were expressed as variance partition coefficient (VPC) and percentage change in variance (PCV). VPC expresses the proportion of the individual differences in the risk of under-5 deaths (i.e., individual variance) that is at the community level. A VPC different from zero is indicative of significant differences in under-5 mortality risks between mothers and communities. We estimated the PCV to evaluate how much of the variance in the first model is attributable to differences in individual characteristics (Merlo et al. 2007). The significance of the random variation at each level was tested with the Wald test, and *p*-values were based on a chi-squared distribution. The deviance information criterion (DIC) was used as a measure of how well the different models fitted the data. Lower values indicate a good model fit relative to the number of parameters in the model (Spiegelhalter et al. 2002).

## Results

**Figure 1:**
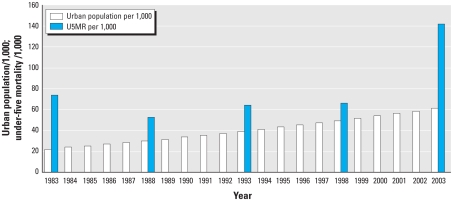
Trends in urban under-5 mortality rates (U5MR) and urban population in Nigeria, 1986–2003. Urban under-5 mortality rates (per 1,000) were directly estimated from the 1990, 1999, and 2003 Nigeria DHS birth history data. Urban population data were from United Nations Department of Economics and Social Affairs (2004).

The urban under-5 mortality rate in Nigeria declined from 74 per 1,000 in the period 1979–1983 to 52 per 1,000 in 1984–1988. It then increased to 142 per 1,000 in the period 1999–2003. Urban population in Nigeria showed a steady increase from about 27,000 in 1986 to about 61,000 in 2003 (urban population here refers to the de facto population living in areas classified as urban according to the criteria used by each area or country) (Figure 1).

Table 1 presents the distribution of the independent variables by UADI. Children in the most disadvantaged UADI quintile (class V) most frequently were male, were of high birth order and medium birth interval (order ≥ 5 and 24–47 months), and had mothers who were younger (24–28 years of age), married, and uneducated, worked as clerical/sales/services/skilled manual employees, and were in the poorest household wealth quintile. On the other hand, a higher proportion of children in the least disadvantaged UADI quintile (class I) were male, were of low birth order and medium birth interval (order 2–4 and 24–47 months), and had mothers who were older (≥ 34 years), married, and educated at the secondary or higher level, worked as clerical/sales/services/skilled manual employees, and were in the richest household wealth quintile.

**Figure 2:**
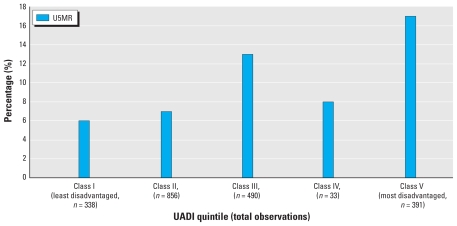
Distribution of under-5 mortality rate (U5MR) according to UADI among participants in the Nigeria Demographic and Health Survey (2003).

Figure 2 illustrates the association between under-5 mortality and UADI and shows that under-5 mortality varied according to urban area disadvantage, with moderate increases in under-5 mortality risk associated with increasing urban area disadvantage. This means that the risks of dying were higher for children of mothers residing in increasingly disadvantaged urban areas.

Table 2 presents the results of the multilevel analysis for the association between urban area disadvantage and under-5 mortality. Model 0 gives an indication of the amount of spatial clustering of under-5 mortality and indicates that the community-level variance is significant (τ = 0.273, *p* = 0.014), whereas the mother-level variance remains nonsignificant, suggesting some clustering of mothers of children with similar risks of under-5 deaths within disadvantaged communities. Results from model 1 indicate a 30% and 50% increase in under-5 deaths among the more disadvantaged children compared with the least disadvantaged UADI quintile. The associations were, however, statistically significant only for class II and class III compared with class I (class II: OR = 1.32; 95% CI, 1.19–1.54; and class III: OR = 1.39; 95% CI, 1.26–1.56). The community-level variation decreased from model 0 but remained significant (τ = 0.129, *p* = 0.063), indicating some clustering of mothers of children with similar risks of death within disadvantaged communities—a compositional effect; that is, the increased risks are explained by the increased risks of the residents who live in that neighborhood. The PCV indicated that 52.7% and 44.9% of the variance in the odds of under-5 mortality across communities and mothers, respectively, were explained by the UADI. Inclusion of child-level characteristics in model 2 did not affect associations of under-5 deaths with children in the more disadvantaged UADI quintiles, but the relative risk was double for children of high birth order after short birth interval (order ≥ 5 and < 24 months) compared with children of intermediate birth order and birth interval (order 2–4 and 24–47 months). The community-level variance decreased further while remaining significant (τ = 0.103, *p* = 0.035), indicating clustering of mothers of children with similar risk factors within disadvantaged communities, a similar compositional effect. The PCV of the odds of under-5 mortality in this model was 20.1% across communities and 24.6% across mothers.

After further adjustment for mother-level characteristics in model 3 relative risks of under-5 deaths increased as the level of disadvantaged UADI quintiles increased. Under-5 deaths among children of mothers in the most disadvantaged UADI quintiles (class V) were double relative to under-5 deaths among children of mothers in the least disadvantaged UADI quintile (class I; OR = 2.14; 95% CI, 1.11–4.12). Furthermore relative risks were significantly increased *a*) for children who were first births (OR = 1.66; 95% CI, 1.04–2.66) or high birth order after short birth interval (order ≥ 5 and < 24 months) (OR = 1.55; 95% CI, 1.01–2.36) compared with intermediate birth order and interval children; *b*) for children of mothers with no education (OR = 2.34; 95% CI, 1.31–3.16) or primary education (OR = 2.00; 95% CI, 1.27–3.13) compared with secondary or higher education; *c*) for children of mothers who were not working (OR = 2.56; 95% CI, 1.03–6.34) versus mothers in professional, technical, or management jobs); and *d*) for children in the poorest versus wealthiest wealth quintile (OR = 1.64; 95% CI, 1.08–2.57). The community-level variance was unchanged from model 2 and still significant (τ = 0.103, *p* = 0.043), indicating clustering of mothers of children with similar risk factors within disadvantaged communities, and also implying a contextual effect that persisted after having accounted for relevant differences between disadvantaged neighborhoods in the characteristics of individual residents. The PCV of the odds of under-5 mortality was 6.8% and 34.7% across communities and mothers, respectively. However, we still found a fairly large amount of “unexplained” variation among communities, which was probably due to other unmeasured individual- and community-level factors. Lower DIC values with successive models indicated that our analytic model was a good fit.

## Discussion

### Trend in urban under-5 mortality

We found that under-5 mortality rate increased with increasing urban population growth in Nigeria (urbanization) between the periods 1979–1983 and 1999–2003. On examination of the association between under-5 mortality and UADI, our findings indicated that under-5 mortality rate increased with increasing levels of urban area disadvantage. Thus, the results of our study, in line with the results of other studies (Eloundou-Enyegue et al. 2000), suggest that with the increasing urban population and the resulting rapid urbanization within the context of poor economic circumstances in Nigeria, an increasing proportion of urban dwellers live in disadvantaged urban neighborhoods with associated increased risks of under-5 deaths.

### Multilevel logistic regression modeling

This study provides evidence that the characteristics of urban areas have a significant association with the risks of under-5 deaths, above and beyond the mother’s SEP. Living in urban neighborhoods that are more socioeconomically disadvantaged thus represents an independent mortality risk factor for children < 5 years of age, which confirms the findings from recent studies (Guidotti and Gitterman 2007; Pongou et al 2006). The increased risk of under-5 deaths among children living in these disadvantaged areas may be explained either directly as a result of living in a deprived neighborhood, as also reported in other studies (Dibben et al. 2006; Pickett and Pearl 2001), or indirectly as a sum of the socioeconomic characteristics of people living within these disadvantaged areas. Among such characteristics, we found that first birth was positively associated with under-5 death. Residing in a disadvantaged urban area may in itself be an important predictor of the survival status of the first child. We found that mothers resident in highly disadvantaged areas were more likely to be younger and of low SEP (uneducated and in the poorest household wealth quintile) than were other mothers. Lack of maternal experience in child care and lack of knowledge of health information may predispose first-born children of younger disadvantaged mothers to increased risks of morbidity and mortality (El-Zanaty and Way 2009). The survival of first births may also be associated with birth spacing and age of the mother at the time of the second birth (Rahman et al. 1996). Moreover, we found that high birth order after short birth interval was associated with increased risks of under-5 deaths, an expected finding shown by other studies (Makepeace and Pal 2006). Preceding birth intervals of 36–59 months have been shown to be optimal for reducing the risk of neonatal mortality (Conde-Agudelo et al. 2006; Rutstein 2005). In addition, birth-to-pregnancy intervals of ≤ 18 months have been associated with the highest risk of neonatal mortality (which reflects a birth-to-birth interval of < 27 months), with the lowest risks estimated for children with birth-to-pregnancy intervals of ≥ 27 months (or birth intervals of > 35 months) (Marston 2006).

Our results indicated that low SEP (primary education or less, unemployment, and being in the poorest wealth quintile) was positively associated with under-5 deaths. This finding is corroborated by those of other urban studies (Giashuddin et al. 2009; Raphael et al. 2003; Schulz et al. 2008; Singh and Kogan 2007; Songsore 2000). Ultimately, it is the multiplicity of socioeconomic factors at both the individual and community levels that shape the survival chances of children in urban environments.

A number of limitations need to be considered in relation to this study. First, defining neighborhoods according to administratively defined boundaries may not always reflect meaningful neighborhood boundaries, especially for area-based measures that characterize the availability of neighborhood socioeconomic characteristics. Such measures may be particularly sensitive to whether people live near neighborhood boundaries (Macintyre et al. 1993). The effect of this nondifferential misclassification of individuals into an inappropriate administrative boundary can generate information biases and reduce the validity of analyses. Second, indices in general are difficult to construct and validate and tend to mask variation in the characteristics that contribute to a score when two or more areas have the same score (Pickett and Pearl 2001).

The strengths of this study are also worth mentioning. First, neighborhood-level socioeconomic characteristics are much more highly correlated than are individual-level socioeconomic factors; hence, the risk of misspecifying the neighborhood-level effect is minimal (Pickett and Pearl 2001). Second, the development of composite indices enable easy handling of several highly correlated neighborhood-level variables and improves statistical efficiency and simplifies the presentation of results. Using several single neighborhood-level measures separately to reflect a single underlying concept such as urban SEP introduces the risk of collinearity and cumbersome results, a point emphasized by previous studies (Pickett and Pearl 2001). Third, the DHS surveys are nationally representative and allow for generalization of the results across the country (Fotso 2006). Fourth, variables in the DHS surveys are defined similarly across countries, and results are therefore comparable across countries (de Walque 2006). Fifth, the advantages of using administrative boundaries are the possibility of comparing any set of data on the same geographic frame, or of presenting complex data in a simple way. Last, further inclusion of individual-level characteristics to the model may have resulted in reduced strength of the association with area disadvantage.

### Policy implications

Several policy implications are therefore inherent from our findings. First, there is a need for accessible and relevant data to better describe and quantify relationships between health outcomes and the urban environment. Second, because most disadvantaged urban neighborhoods are characterized by significant levels of inequality, we do not necessarily support a policy that concentrates only on the most deprived areas of low- and middle-income countries such as Nigeria, because interventions resulting from policies that focus solely on priority areas risk excluding a major proportion of mothers and children who might otherwise have benefited from resulting interventions and widening such inequalities. Hence, there is a need to focus on inequality-reduction measures. Third, there is a need for policies to promote the optimal birth interval of 36–59 months, which has been repeatedly observed to reduce risk of neonatal or child mortality (Setty-Venugopal 2002), or a birth-to-pregnancy interval of 24 months (Marston 2006).

## Conclusion

We found that urban-area disadvantage was independently associated with the risk of under-5 deaths even after controlling for individual child- and mother-level demographic and socioeconomic characteristics. The existence of significant risk of under-5 deaths at both the individual and community levels underscores the need not only to tailor interventions aiming at the community level (the disadvantaged neighborhoods) but also to focus strategies implemented at the individual level. Community- or neighborhood-level strategies could aim to counter adverse environmental conditions of deprived areas, such as the sustainable development of urban household amenities and community infrastructure, improved water supply, improved maternal literacy, education, and employment, and other neighborhood socioeconomic upliftment strategies in these deprived communities. Increased relative risks associated with first births and high-order births after short preceding birth interval emphasizes the need for strategies that promote optimal birth-to-birth intervals and enhanced health-seeking behavior of mothers in these disadvantaged areas, especially young uneducated mothers. Significant variation among communities found in this study stresses the need for further studies on possible unmeasured community-level determinants of under-5 mortality in disadvantaged urban areas.

## Correction

In Table 2, the variance values were incorrect in the manuscript originally published online. They have been corrected here.

## Figures and Tables

**Table 1:** Sociodemographic characteristics of the urban population according to UADI [*n* (%)].

Characteristic	Class I (least disadvantaged; *n* = 338)	Class II (*n* = 856)	Class III (*n* = 490)	Class IV (*n* = 43)	Class V (most disadvantaged; *n* = 391)	Total (*n* = 2,118)
Child’s sex						
Male	172 (51)	424 (49)	238 (49)	24 (56)	218 (56)	1,076
Female	166 (49)	432 (51)	252 (51)	19 (44)	173 (44)	1,042
Child’s birth order, birth interval						
First birth (order 1)	71 (21)	247 (29)	83 (17)	11 (26)	52 (13)	464 (22)
Order 2–4, < 24 months	46 (13)	89 (10)	36 (7)	2 (5)	41 (10)	214 (10)
Order 2–4, 24–47 months	104 (31)	241 (28)	96 (20)	17 (39)	83 (21)	541 (26)
Order 2–4, ≥ 48 months	41 (12)	74 (9)	46 (9)	1 (2)	27 (7)	189 (9)
Order ≥ 5, < 24 months	13 (4)	24 (3)	40 (8)	2 (5)	49 (13)	128 (6)
Order ≥ 5, 24–47 months	47 (14)	129 (15)	148 (30)	6 (14)	101 (26)	431 (20)
Order ≥ 5, ≥ 48 months	16 (5)	52 (6)	41 (8)	4 (9)	38 (10)	151 (7)
Mother’s age (years)						
15–18	1 (0)	27 (3)	22 (4)	3 (7)	23 (6)	76 (4)
19–23	29 (9)	171 (20)	88 (18)	9 (22)	45 (11)	342 (16)
24–28	100 (29)	283 (33)	126 (26)	11 (25)	123 (32)	643 (30)
29–33	97 (29)	188 (22)	97 (20)	11 (25)	87 (22)	480 (23)
≥ 34	111 (33)	187 (22)	157 (32)	9 (21)	113 (29)	577 (27)
Mother’s marital status						
Single	6 (2)	26 (3)	2 (0)	12 (28)	5 (1)	51 (3)
Married	327 (97)	783 (91)	464 (95)	30 (70)	356 (91)	1,960 (92)
Divorced	5 (1)	47 (6)	24 (5)	1 (2)	30 (8)	107 (5)
Mother’s education						
No education	1 (0)	37 (4)	400 (82)	13 (30)	292 (75)	743 (35)
Primary	71 (21)	327 (38)	55 (11)	12 (28)	68 (17)	533 (25)
Secondary or higher	266 (79)	492 (58)	35 (7)	18 (42)	31 (8)	842 (40)
Mother’s occupation						
Not working	45 (14)	313 (37)	187 (38)	19 (44)	136 (35)	700 (33)
Clerical/sales/services/skilled manual	231 (68)	479 (56)	293 (60)	14 (33)	249 (64)	1,266 (60)
Professional/technician/management	62 (18)	64 (7)	10 (2)	10 (23)	6 (1)	152 (7)
Wealth index						
Poorest	0 (0)	0 (0)	3 (1)	10 (23)	107 (27)	120 (6)
Poorer	0 (0)	0 (0)	7 (1)	3 (7)	203 (52)	213 (10)
Middle	1 (0)	97 (11)	162 (33)	21 (49)	70 (18)	351 (16)
Richer	24 (7)	356 (42)	272 (56)	9 (21)	11 (3)	672 (32)
Richest	313 (93)	403 (47)	46 (9)	0 (0)	0 (0)	762 (36)

**Table 2:** Multilevel logistic regression models of urban area disadvantage and under-5 mortality [OR (95% CI)].

Characteristic	Model 0 (empty)	Model 1 (UADI)	Model 2 (child level)	Model 3 (mother level)
Fixed effects				
UADI				
Class I (least disadvantaged)		1	1	1
Class II		1.32 (1.19–1.54)	1.32 (0.19–1.55)	1.72 (0.91–3.29)
Class III		1.39 (1.26–1.56)	1.38 (0.26–1.56)	1.78 (1.17–2.70)
Class IV		1.76 (0.52–1.81)	1.76 (0.52–2.11)	2.03 (1.04–3.97)
Class V (most disadvantaged)		1.51 (0.65–1.72)	1.49 (0.14–1.65)	2.14 (1.11–4.12)

Child’s sex				
Male			1	1
Female			1.04 (0.78–1.39)	1.02 (0.76–1.36)

Child’s birth order, birth interval				
First birth (order 1)			1.40 (0.91–2.13)	1.66 (1.04–2.66)
Order 2–4, < 24 months			1.05 (0.60–1.84)	1.07 (0.61–1.89)
Order 2–4, 24–47 months			1	1
Order 2–4, ≥ 48 months			0.76 (0.40–1.44)	0.65 (0.34–1.27)
Order ≥ 5, < 24 months			2.17 (1.21–3.88)	1.55 (1.01–2.36)
Order ≥ 5, 24–47 months			1.16 (0.75–1.79)	0.81 (0.49–1.35)
Order ≥ 5, ≥ 48 months			0.76 (0.39–1.49)	0.51 (0.24–1.07)

Mother’s marital status				
Single				0.67 (0.20–2.30)
Married				1
Divorced				1.57 (0.73–3.37)

Mother’s age (years)				
15–18				0.84 (0.39–1.80)
19–23				0.81 (0.50–1.31)
24–28				1
29–33				1.08 (0.69–1.69)
≥ 34				1.53 (0.94–2.47)

Mother’s education				
No education				2.34 (1.31–3.16)
Primary				2.00 (1.27–3.13)
Secondary or higher				1

Mother’s occupation				
Not working				2.56 (1.03–6.34)
Clerical/sales/services/skilled manual				1.53 (0.63–3.69)
Professional/technical/management				1

Wealth index				
Poorest				1.64 (1.08–2.57)
Poorer				1.60 (0.68–3.76)
Middle				1.50 (0.85–2.64)
Richer				1.01 (0.62–1.62)
Richest				1

Community level				
Variance (*p*-value)	0.273 (0.111)	0.129 (0.063)	0.103 (0.043)	0.097 (0.051)
VPC	7.4	3.7	3.0	2.8
Explained variation (PCV) (%)	Reference	52.7	20.1	6.8

Mother level				
Variance (*p*-value)	0.118 (0.334)	0.065 (0.107)	0.049 (0.091)	0.032 (0.020)
VPC	3.2	1.9	1.4	0.9
Explained variation (PCV) (%)	Reference	44.9	24.6	34.7
DIC	1,398	1,375	1,365	1,287

Data from Nigeria Demographic and Health Survey (2003).
